# Clinical evaluation of a multitarget fecal immunochemical test‐sDNA test for colorectal cancer screening in a high‐risk population: a prospective, multicenter clinical study

**DOI:** 10.1002/mco2.345

**Published:** 2023-08-12

**Authors:** Ye‐Ting Hu, Xiao‐Feng Chen, Chun‐Bao Zhai, Xiao‐Tian Yu, Gang Liu, Zhi‐Guo Xiong, Zi‐Qiang Wang, San‐Jun Cai, Wen‐Cai Li, Xiang‐Xing Kong, Qian Xiao, Cai‐Hua Wang, Zhi‐Hua Tao, Li‐Yun Niu, Jian‐Long Men, Qing Wang, Shao‐Zhong Wei, Jun‐Jie Hu, Ting‐Han Yang, Jun‐Jie Peng, Guo‐Zhong Jiang, Ning Lv, Yi‐You Chen, Shu Zheng, Yan‐Hong Gu, Ke‐Feng Ding

**Affiliations:** ^1^ Department of Colorectal Surgery and Oncology (Key Laboratory of Cancer Prevention and Intervention, China National Ministry of Education, Key Laboratory of Molecular Biology in Medical Sciences, Zhejiang Province, China) The Second Affiliated Hospital, Zhejiang University School of Medicine Hangzhou Zhejiang China; ^2^ Department of Oncology The First Affiliated Hospital with Nanjing Medical University (Jiangsu Province Hospital) Nanjing China; ^3^ Department of Anorectal Surgery Shanxi Provincial People's Hospital Taiyuan China; ^4^ Hangzhou New Horizon Health Technology Co., Ltd. Hangzhou China; ^5^ Department of General Surgery Tianjin Medical University General Hospital Tianjin China; ^6^ Department of Gastrointestinal surgery Hubei Cancer Hospital Wuhan China; ^7^ Colorectal Cancer Medical Research Center of Hubei Wuhan China; ^8^ Department of Gastrointestinal Surgery West China Hospital of Sichuan University Chengdu China; ^9^ Department of Colorectal Surgery Fudan University Shanghai Cancer Center Shanghai China; ^10^ Department of Pathology The First Affiliated Hospital of Zhengzhou University Zhengzhou China; ^11^ Department of Gastroenterology The Second Affiliated Hospital of Zhejiang University School of Medicine Hangzhou China; ^12^ Department of Clinical Laboratory The Second Affiliated Hospital of Zhejiang University School of Medicine Hangzhou China; ^13^ Cancer Institute, Key Laboratory of Cancer Prevention and Intervention, Ministry of Education, The Second Affiliated Hospital Zhejiang University School of Medicine Hangzhou China; ^14^ Center for Medical Research and Innovation in Digestive System Tumors Hangzhou China; ^15^ Zhejiang Provincial Clinical Research Center for CANCER Hangzhou China; ^16^ Cancer Center of Zhejiang University Hangzhou China

**Keywords:** colorectal cancer, FIT‐sDNA test, high‐risk population, risk triaging modality

## Abstract

Colorectal cancer (CRC) is a major malignancy threatening the health of people in China and screening could be effective for preventing the occurrence and reducing the mortality of CRC. We conducted a multicenter, prospective clinical study which recruited 4,245 high‐risk CRC individuals defined as having positive risk‐adapted scores or fecal immunochemical test (FIT) results, to evaluate the clinical performance of the multitarget fecal immunochemical and stool DNA (FIT‐sDNA) test for CRC screening. Each participant was asked to provide a stool sample prior to bowel preparation, and FIT‐sDNA test and FIT were performed independently of colonoscopy. We found that 186 (4.4%) were confirmed to have CRC, and 375 (8.8%) had advanced precancerous neoplasia among the high CRC risk individuals. The sensitivity of detecting CRC for FIT‐sDNA test was 91.9% (95% CI, 86.8–95.3), compared with 62.4% (95% CI, 54.9–69.3) for FIT (*P* < 0.001). The sensitivity for detecting advanced precancerous neoplasia was 63.5% (95% CI, 58.3–68.3) for FIT‐sDNA test, compared with 30.9% (95% CI, 26.3–35.6) for FIT (*P* < 0.001). Multitarget FIT‐sDNA test detected more colorectal advanced neoplasia than FIT. Overall, these findings indicated that in areas with limited colonoscopy resources, FIT‐sDNA test could be a promising further risk triaging modality to select patients for colonoscopy in CRC screening.

## Graphical abstract

We conducted a multicenter, prospective clinical study which recruited 4,245 high‐risk CRC individuals to evaluate the clinical performance of the multitarget fecal immunochemical and stool DNA (FIT‐sDNA) test for CRC screening. We found that the FIT‐sDNA test detected more colorectal advanced neoplasia than FIT. It indicated that in areas with limited colonoscopy resources, the FIT‐sDNA test could be a promising further risk triaging modality to select patients for colonoscopy in CRC screening.

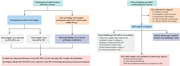

## 1 INTRODUCTION

Colorectal cancer (CRC) is ranked second in incidence and fourth in mortality among all cancers in China,^1^ and accumulating evidence suggests that screening is effective for preventing the occurrence and reducing the mortality of CRC.^1^, ^2^, ^3^, ^4^, ^5^, ^6^, ^7^ However, the population between 40 and 74 years old is over 600 million in China, which far exceeds the reported annual colonoscopy capability of 5.8 million,^8^ making it unfeasible to apply direct colonoscopy for CRC screening. Fecal immunochemical test (FIT) and/or risk‐adapted scoring such as high‐risk factor questionnaire (HRFQ),^4^, ^9^, ^10^ Asia‐Pacific Colorectal Screening (APCS) score,^11^, ^12^ and modified APCS score have been used for preliminary CRC screening.^13^, ^14^, ^15^ Individuals who have positive risk‐adapted scores or FIT results are defined as high‐risk CRC individuals and are referred to follow‐up colonoscopy. This two‐stage screening strategy has been performed for decades but has achieved limited success due to high preliminary screening positive rate, low CRC/adenoma detection rate, and poor colonoscopy compliance.^16^, ^17^ Therefore, a new test method for further risk triage in individuals who are identified as high‐risk of CRC by preliminary screening is urgently needed. Individuals with positive risk‐adapted score or a FIT result may complete an additional test to improve the screening effectiveness.

The multitarget fecal immunochemical and stool DNA (FIT‐sDNA) test is a novel screening method recommended by United States (US) guidelines for CRC screening in average‐risk populations.^6^, ^18^ This method detects cancer‐related genetic and epigenetic alterations contained in the exfoliated colonic epithelial cells, and it is combined with a specific hemoglobin (Hb) test to derive a final composite score using a logistic‐regression algorithm. A large‐scale, prospective clinical study in an average‐risk US population has demonstrated that the FIT‐sDNA test has higher sensitivity in detecting CRC and advanced precancerous neoplasia than FIT.^19^ To date, however, there is no large‐scale prospective study to evaluate the clinical performance of the FIT‐sDNA test for CRC screening for individuals with high‐risk profiles.

ColoClear^®^ is a newly developed FIT‐sDNA test that consists of molecular assays for KRAS mutation, aberrant methylation of the BMP3 promoter region, and aberrant methylation of the NDRG4 promoter region as well as a hemoglobin test. The methylation target sites were based on Asian population data, and the stool‐processing method and logistic‐regression algorithm were further optimized to improve the sensitivity.

In the present study, we report the outcome of the prospective cohort from the multicenter clinical trial that targeted high‐risk CRC individuals identified by FIT and/or HRFQ. The primary objective was to assess the clinical performance of the FIT‐sDNA test for detecting CRC in high‐risk individuals, and the secondary objective was to investigate whether the FIT‐sDNA test is superior to the conventional FIT in detecting advanced precancerous neoplasia. Our results provide a promising screening modality for further risk triage in high‐risk CRC individuals from preliminary CRC screening.

## 2 RESULTS

### 2.1 Study population

**FIGURE 1:**
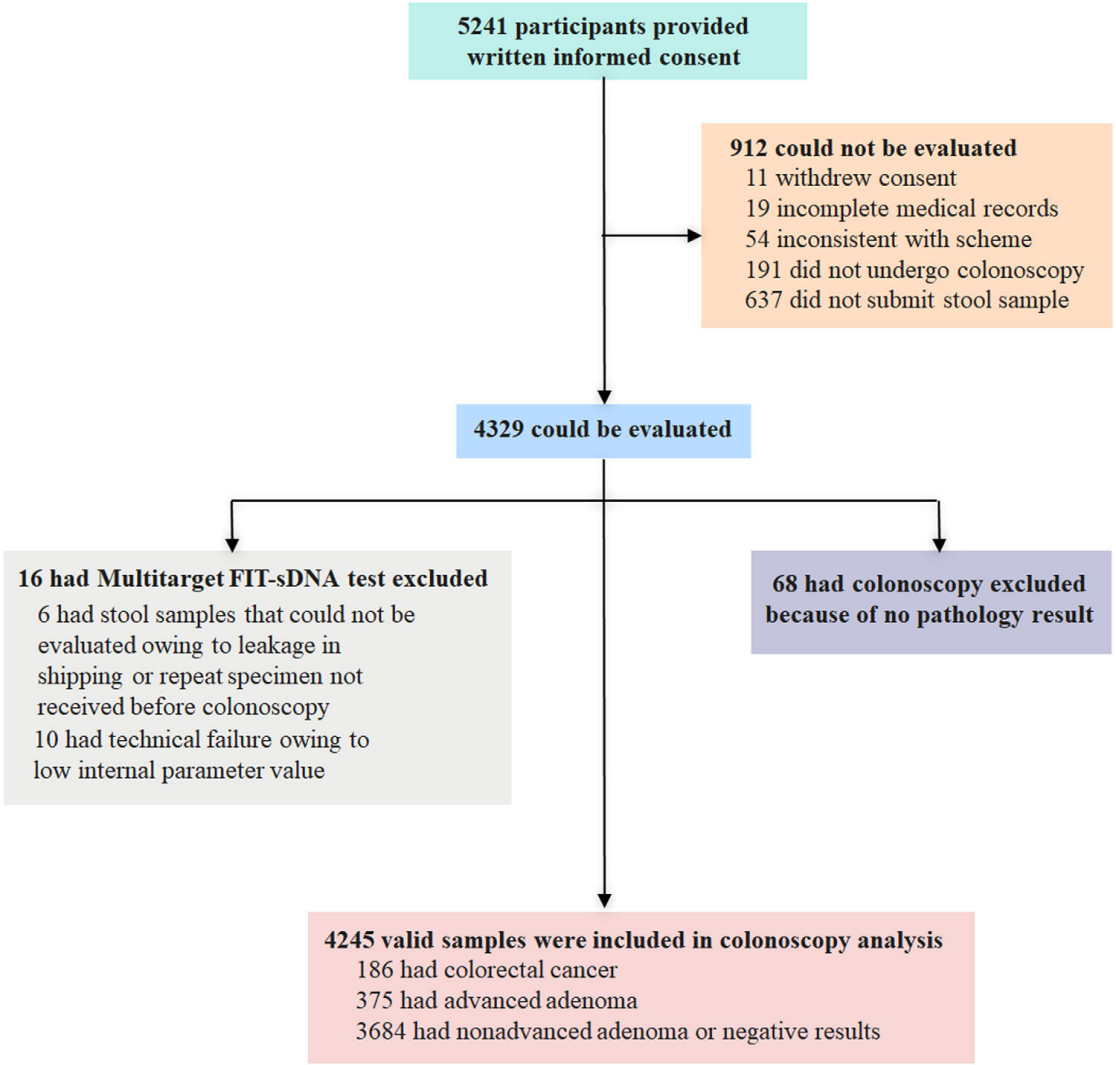
Study flow chart. Among the 4,245 evaluable participants, 186 participants were diagnosed with CRC (4.4% prevalence), and 375 participants were found to have advanced precancerous neoplasia (8.8% prevalence). Despite the fact that 55.7% of the overall participants were females, 104 (55.9%) participants who had CRC and 241 (64.3%) participants who had advanced precancerous neoplasia were males (Table 1). Among the participants with confirmed CRC, 85 participants had early‐stage CRC (Stages I to II), and 140 participants had CRC Stage I to III. CRC was found more frequently in the distal section of the colon than the proximal section (154 vs. 32). Details of the clinical findings are shown in Table S1 (in the Appendix, Supporting information). CRC, colorectal cancer.

Between September 2018 and November 2019, a total of 5,241 participants were enrolled from eight tertiary‐level hospitals, and 4,245 (80.1%) participants were fully evaluated for the final analysis (Figure 1). Among the 4,245 participants, 376 (8.9%) participants had family history of CRC, 182 (4.3%) participants had a history of positive fecal occult blood test (FOBT), 3,632 (85.6%) participants had clinical symptoms or conditions, and 55 (1.3%) participants had mixed indications (Table 1). The median participant age was 58 years old, and the majority (95%) were between 40 and 69 years old. Overall, 2,366 (55.7%) of participants were females and 1879 (44.3%) were males. The characteristics of the evaluable study population were summarized in Table 1.

**TABLE 1:** Characteristics of evaluable study population.

Characteristics	All (*N* = 4245)	CRC (*N* = 186)	Advanced precancerous neoplasia^*^ (*N* = 375)	Nonadvanced adenoma (*N* = 672)	All non‐neoplastic lesions (*N* = 1444)	Negative colonoscopy results (*N* = 1568)
Gender, *n* (%)						
Males	1879 (44.3)	104 (55.9)	241 (64.3)	358 (53.3)	653 (45.2)	523 (33.4)
Females	2366 (55.7)	82 (44.1)	134 (35.7)	314 (46.7)	791 (54.8)	1045 (66.6)
Median age, *y* (range)	58 (40–74)	62 (42–74)	65 (40–73)	62 (40–74)	57 (40–74)	55 (40–73)
Age group, *n* (%)						
40–49	1233 (29.0)	30 (16.1)	77 (20.5)	138 (20.5)	423 (29.3)	565 (36.0)
50–59	1586 (37.4)	55 (29.6)	116 (30.9)	251 (37.4)	543 (37.6)	621 (39.6)
60–69	1212 (28.6)	78 (41.9)	151 (40.3)	233 (34.7)	408 (28.3)	342 (21.8)
70–74	214 (5.0)	23 (12.4)	31 (8.3)	50 (7.4)	70 (4.8)	40 (2.6)
History of positive FOBT, *n* (%)	182 (4.3)	9 (4.8)	21 (5.6)	37 (5.5)	57 (3.9)	58 (3.7)
Family history of CRC, *n* (%)	376 (8.9)	2 (1.1)	36 (9.6)	60 (8.9)	117 (8.1)	161 (10.3)
Clinical symptoms or conditions, *n* (%)	3632 (85.6)	172 (92.5)	314 (83.7)	570 (84.8)	1250 (86.6)	1326 (84.6)
Mixed indications^†^, *n* (%)	55 (1.3)	3 (1.6)	4 (1.1)	5 (0.7)	20 (1.4)	23 (1.5)

* Advanced precancerous neoplasia included advanced adenomas, SSA/P and TSA ≥1 cm.

^†^ Mixed indications included participants with two or more indications of family history of CRC, history of positive FOBT, and clinical symptoms or conditions.

Abbreviations: CRC, colorectal cancer; FOBT, fecal occult blood test; SSA, sessile serrated adenoma; TSA, traditional serrated adenoma.

### 2.2 The fecal immunochemical test‐sDNA test showed high sensitivity of detecting colorectal advanced neoplasia

**FIGURE 2:**
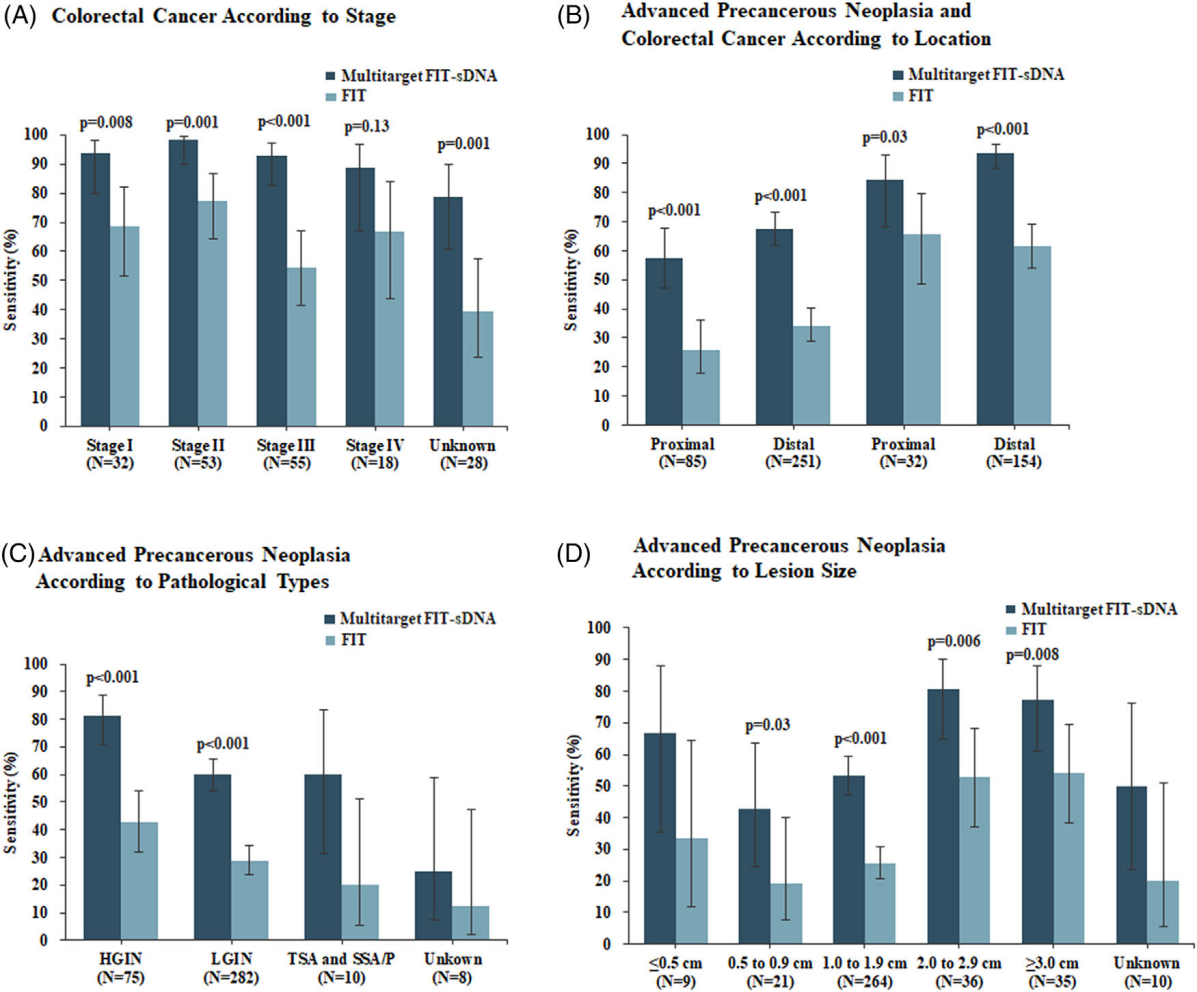
Sensitivity of the multitarget FIT‐sDNA test and the commercial FIT according to the CRC and advanced precancerous neoplasia subgroups. (A) Sensitivities of the FIT‐DNA test and FIT for the detection of CRC according to the tumor stage. (B) CRC and advanced precancerous neoplasia were detected according to the location in the colon. (C) Sensitivities of the FIT‐DNA test and FIT for the detection of advanced adenoma with HGIN, LGIN, TSA, and SSA/P ≥1 cm. (D) Sensitivities of the FIT‐DNA test and FIT according to neoplasia size. CRC, colorectal cancer; FIT, fecal immunochemical test; SSA, sessile serrated adenoma; TSA, traditional serrated adenoma.

One hundred eigty‐six participants were diagnosed as CRC, in which 32 (17.2%) were stage I CRC and 53 (28.5%) were stage II CRC. Besides, distal colorectal cancers (< 60 cm, left hemicolon) accounted for the majority of CRC (154 of 186; Table S1). The FIT‐sDNA test detected 171 of 186 CRC, and the corresponding sensitivity was 91.9% (95% CI, 86.3–95.3) (Table 2). The sensitivities for Stage I and II CRC were 93.8% and 98.1%, respectively (Figure 2A). There was no significant difference in sensitivity according to participants’ CRC tumor node metastasis (TNM) stage or tumor location (Figure 2A,B). The FIT‐sDNA test identified 238 out of 375 participants who had advanced precancerous neoplasia with a sensitivity of 63.5% (95% CI, 58.3–68.3; Table 2). The sensitivity for detecting adenomas with high‐grade intraepithelial neoplasia was higher than that for low‐grade intraepithelial neoplasia (81.3% and 59.9%, respectively, *P* < 0.001), as demonstrated in Figure 2C. Higher sensitivity was observed when the lesion size increased from 0.5 to 2.9 cm (*P* = 0.029 for the comparison between 0.5 and 2.9 cm lesion size), but there was no significant difference in sensitivity among lesion sizes ranging from 2.0 to 2.9 cm and those larger than 3 cm (Figure 2D). In different age subgroups, the sensitivities of the FIT‐sDNA test for detecting advanced precancerous neoplasia among participants aged 40 to 49, 50 to 59, 60 to 69, and 70 to 74 years were 55.8% (95% confidence interval (CI), 44.1–67.0), 65.5% (95% CI, 56.1–73.9), 66.2% (95% CI, 58.0–73.6), and 61.3% (95% CI, 42.3–77.6), respectively (Table 3). The overall sensitivity of the FIT‐sDNA test for detecting advanced colorectal neoplasia was 72.9% (95% CI, 69.0–76.5; Table 2).

**TABLE 2:** Sensitivity and specificity of the multitarget FIT‐sDNA test and FIT for the most advanced findings from colonoscopies.

Most advanced finding	Colonoscopy (*N* = 4245)	FIT‐sDNA test (*N* = 4245)		FIT (*N* = 4245)	
		Positive results	Sensitivity (95% CI)	Positive results	Sensitivity (95% CI)
	no.	no.	%	no.	%
CRC	186	171	91.9 (86.8–95.3)	116	62.4 (54.9–69.3)
Advanced precancerous neoplasia	375	238	63.5 (58.3–68.3)	116	30.9 (26.3–35.6)
Advanced colorectal neoplasia^*^	561	409	72.9 (69.0–76.5)	232	41.4 (37.3–45.6)
Nonadvanced adenoma	672	106	15.8 (13.1–18.8)	49	7.3 (5.5–9.6)

			Specificity (95% CI)		Specificity (95% CI)
All nonadvanced precancerous neoplasia, non‐neoplastic findings, and negative colonoscopy results	3684	476	87.1 (85.9–88.1)	189	94.9 (94.2–95.6)
Negative colonoscopy results	1568	152	90.3 (88.7–91.7)	44	97.2 (96.2–97.9)

* Advanced colorectal neoplasia, including CRC and advanced adenoma.

Abbreviations: CI, confidence intervals; CRC, Colorectal cancer; FIT, fecal immunochemical test.

**TABLE 3:** Sensitivity and specificity of the multitarget FIT‐sDNA test and FIT for the most advanced findings from colonoscopies in different age groups.

Age		CRC			Advanced precancerous neoplasia			All nonadvanced precancerous neoplasia, non‐neoplastic findings, and negative colonoscopy results		
			FIT‐sDNA test sensitivity (95% CI)	FIT sensitivity (95% CI)		FIT‐sDNA test sensitivity (95% CI)	FIT sensitivity (95% CI)		FIT‐sDNA test specificity (95% CI)	FIT specificity (95% CI)
*Subgroup*	*no*.	*no*.	*%*	*%*	*no*.	*%*	*%*	*no*.	*%*	*%*
All	4245	186	91.9 (86.8–95.3)	62.4 (54.9–69.3)	375	63.5 (58.3–68.3)	30.9 (26.3–35.9)	3684	87.1 (85.9–88.1)	94.9 (94.1–95.5)
40∼49	1233	30	93.3 (76.5–98.8)	60.0 (40.8–76.8)	77	55.8 (44.1–67.0)	29.9 (20.2–41.5)	1126	89.6 (87.6–91.3)	95.6 (94.2–96.7)
50∼59	1586	55	89.1 (77.1–95.5)	60.0 (45.9–72.7)	116	65.5 (56.1–73.9)	27.6 (19.9–36.8)	1415	89.1 (87.3–90.7)	96.0 (94.8–96.9)
60∼69	1212	78	94.9 (86.7–98.3)	64.1 (52.4–74.4)	151	66.2 (58.0–73.6)	35.1 (27.6–43.3)	983	82.3 (79.7–84.6)	92.8 (90.9–94.3)
70∼74	214	23	87.0 (65.3–96.6)	65.2 (42.8–82.8)	31	61.3 (42.3–77.6)	25.8 (12.5–44.9)	160	80.6 (73.5–86.3)	92.5 (87.0–95.9)

Abbreviations: CI, confidence intervals; CRC, Colorectal cancer; FIT, fecal immunochemical test.

For the 1,568 participants who had negative colonoscopy results, the specificity of the FIT‐sDNA test was 90.3% (95% CI, 88.7–91.7). For 3,684 participants who had no findings of advanced colorectal neoplasia, the specificity was 87.1% (95% CI, 85.9–88.1; Table 2). Moreover, the specificities of the FIT‐sDNA test in the 40 to 49, 50 to 59, 60 to 69, and 70 to 79 years old group were 89.6% (95% CI, 87.6–91.3), 89.1% (95% CI, 87.3–90.7), 82.3% (95% CI, 79.7–84.6), and 80.6% (95% CI, 73.5–86.3; Table 3).

Receiver operating characteristic (ROC) analysis revealed that the area under the curve (AUC) values of the FIT‐sDNA test for CRC and advanced colorectal neoplasia were 0.916 (95% CI, 0.893–0.939) and 0.827 (95% CI, 0.805–0.848), respectively (Figure S1).

### 2.3 The sensitivity of the fecal immunochemical test was inferior to the FIT‐sDNA test

The FIT identified 116 out of 181 participants with confirmed CRC with a sensitivity of 62.4% (95% CI, 54.9–69.3; Table 2), which was lower than that of the FIT‐sDNA test (*P* < 0.001). The sensitivities of the FIT for Stages I and II CRC were 68.8% and 77.3%, respectively (Figure 2A). Compared to the FIT‐sDNA test, the sensitivity of the FIT was significantly lower for CRC TNM Stages I–III (Figure 2A), as well as for CRC located proximal or distal of the colon (Figure 2B).

The FIT detected 116 out of 375 participants who had advanced precancerous neoplasia with a sensitivity of 30.9% (95% CI, 26.3–35.6; Table 2). Compared to the FIT‐sDNA test, the FIT had a lower sensitivity for detecting advanced precancerous neoplasia. The sensitivity of the FIT for detecting adenomas with high‐grade intraepithelial neoplasia was higher than that for detecting adenomas with low‐grade intraepithelial neoplasia, but the difference did not reach statistical significance (*P* = 0.062 for the comparison between high‐ and low‐grade intraepithelial neoplasia; Figure 2C). The sensitivity of the FIT for detecting traditional serrated adenoma (TSA) and sessile serrated adenoma/polyp (SSA/P) was 25% compared to 62.5% of the FIT‐sDNA test. For lesions larger than 2 cm, the sensitivity was significantly increased (Figure 2D). In addition, the sensitivities of the FIT for detecting advanced precancerous neoplasia among participants aged 40 to 49, 50 to 59, 60 to 69, and 70 to 74 years were 29.9% (95% CI, 20.2–41.5), 27.6% (95% CI, 19.9–36.8), 35.1% (95% CI, 27.6–43.3), and 25.8% (95% CI, 12.5–44.9), respectively (Table 3). Overall, the sensitivity of the FIT was inferior to the FIT‐sDNA test for detecting CRC and advanced precancerous neoplasia.

For 3,684 participants who had no findings of advanced colorectal neoplasia and 1,568 participants who had negative colonoscopy results, the specificities of the FIT were 94.9% (95% CI, 94.2–95.6) and 97.2% (95% CI, 96.2–97.9), respectively (Table 2). Furthermore, there was no obvious difference of specificity among age subgroups, with 95.6% (95% CI, 94.2–96.7) in the 40‐ to 49‐ year‐ old group, 96.0% (95% CI, 94.8–96.9) in the 50‐ to 59‐ year‐ old group, 92.8% (95% CI, 90.9–94.3) in the 60‐ to 69‐ year‐ old group, and 92.5% (95% CI, 87.0–95.9) in the 70‐ to 79‐ year‐ old group (Table 3). In addition, the specificities of the FIT in the history of positive fecal occult blood test (FOBT) subgroup, family history of CRC subgroup, clinical symptoms or conditions subgroup, and mixed indications subgroup were 88.8% (95% CI, 82.8–92.9), 97.6% (95% CI, 95.4–98.9), 94.8% (95% CI, 94.0‐95.6), and 92.2% (95% CI, 80.3–97.5, Table S3). Overall, the specificities of the FIT were superior to those of the FIT‐sDNA test (*P* < 0.001).

### 2.4 The FIT‐sDNA test exhibited the important performance value in CRC screening

To evaluate the performance of the FIT‐sDNA test for CRC screening, the number of tests that needed to detect one CRC and one advanced precancerous neoplasia in the study population was calculated in Table S2. To detect one CRC at any stage, the number of colonoscopies, FIT‐sDNA tests, and FITs needed was 23 (95% CI, 20–26), 25 (95% CI, 21–29), and 37 (95% CI, 30–43), respectively. In order to find Stages I to III CRC, the number of colonoscopies, FIT‐sDNA tests, and FITs needed was 39 (95% CI, 32–48), 41 (95% CI, 33–50), and 58 (95% CI, 45–71), respectively. For advanced precancerous neoplasia screening, the number of required colonoscopies, FIT‐sDNA tests, and FITs were 11 (95% CI, 10–13), 18 (95% CI, 16–20), and 37 (95% CI, 30–43), respectively. These data suggested that the performance of the FIT‐sDNA test in detecting CRC and advanced precancerous neoplasia was superior to the FIT in the high‐risk population.

Table 4 showed the detailed data analysis of FIT‐sDNA test and FIT among groups with different colonoscopy findings, and the positive prediction value (PPV) and negative prediction value (NPV) were shown in Table 5. The PPVs of the FIT‐sDNA test for CRC, advanced precancerous neoplasia, and advanced colorectal neoplasia were 19.3% (95% CI, 16.8–22.1), 26.9% (95% CI, 24.0–30.0), and 46.2% (95% CI, 42.9–49.6), respectively, while the NPVs were 99.6% (95% CI, 99.2–99.7), 95.9% (95% CI, 95.2–96.6), and 95.5% (95% CI, 94.7–96.1), respectively (Table 5). The PPV of the FIT‐sDNA test for CRC and advanced colorectal neoplasia was 4.4 and 3.5 times compared to the prevalence rate (4.4% for CRC and 13.2% for advanced colorectal neoplasia) in the study population, respectively.

**TABLE 4:** Colonoscopy finding for colorectal cancer undergoing screening with colonoscopy, multitarget FIT‐sDNA test, and FIT in study population.

Colonoscopy finding	Persons with finding	FIT‐sDNA Test		FIT	
		Positive results (*N* = 885)	Negative results (*N* = 3360)	Positive results (*N* = 421)	Negative results (*N* = 3824)
		*n* (%)	*n* (%)	*n* (%)	*n* (%)
Colorectal cancer	186	171 (19.3)	15 (0.4)	116 (27.6)	70 (1.8)
Advanced precancerous neoplasia	375	238 (26.9)	137 (4.1)	116 (27.6)	259 (6.8)
Advanced Colorectal Neoplasia	561	409 (46.2)	152 (4.5)	232 (55.1)	329 (8.6)
Nonadvanced adenoma	672	106 (12.0)	566 (16.8)	49 (11.6)	623 (16.3)
All nonadvanced precancerous neoplasia, non‐neoplastic findings, and negative results on colonoscopy	3684	476 (53.8)	3208 (95.5)	189 (44.9)	3495 (91.4)

Abbreviation: FIT, fecal immunochemical test.

**TABLE 5:** Positive predictive value and negative predictive value of FIT‐sDNA test and FIT.

		FIT‐sDNA		FIT	
Clinical findings	Prevalence	PPV (95% CI)	NPV (95% CI)	PPV (95% CI)	NPV (95% CI)
Colorectal cancer	4.4	19.3 (16.8–22.1)	99.6 (99.2–99.7)	27.6 (23.4–32.1)	98.2 (97.7–98.6)
Advanced precancerous neoplasia	8.8	26.9 (24.0–30.0)	95.9 (95.2–96.6)	27.6 (23.4–32.1)	93.2 (92.4–94.0)
Advanced colorectal neoplasia	13.2	46.2 (42.9–49.6)	95.5 (94.7–96.1)	55.1 (50.2–59.9)	91.4 (90.5–92.3)

Abbreviations: CI, confidence intervals; FIT, fecal immunochemical test; NPV, negative prediction value; PPV, positive prediction value.

The PPVs of the FIT for CRC, advanced precancerous neoplasia, and advanced colorectal neoplasia were 27.6% (95% CI, 23.4–32.1), 27.6% (95% CI, 23.4–32.1), and 55.1% (95% CI, 50.2–59.9), respectively, and the NPVs were 98.2% (95% CI, 97.7–98.6), 93.2% (95% CI, 92.4–94.0), and 91.4% (95% CI, 90.5–92.3), respectively (Table 5). Compared to the FIT‐sDNA test, the FIT had a slightly higher PPV but lower NPV for advanced colorectal neoplasia.

Using the risk triage strategy in the high‐risk population, individuals with positive test results would be referred to immediate follow‐up colonoscopy. In a hypothetical condition that the study population adopts this risk triage strategy, 885 (20.8%) would have positive FIT‐sDNA test results and subjected to follow‐up colonoscopy. Moreover, 409 would be considered true positives, in which advanced colorectal neoplasia is defined as “true finding.” In the 3,360 negative test results, 15 CRC and 137 advanced precancerous neoplasia would have been missed and thus defined as false negatives (Table 4). For comparison, 421 (9.9%) would have positive FIT results with 232 true positives, while 70 CRC and 259 advanced precancerous neoplasia cases would be considered false negatives. Further risk triage with the FIT‐sDNA test would require 464 more colonoscopies than the FIT group, but the FIT‐sDNA test would have detected 177 more advanced colorectal neoplasia cases. The true positive rate for the additional colonoscopy subgroup would be 38.1%, which is 2.8 times the prevalence rate. Overall, these results indicated the important performance value of the FIT‐sDNA test in further risk stratification of CRC screening.

## 3 DISCUSSION

The present study is the first prospective multicenter clinical study to evaluate the performance of the FIT‐sDNA test and conventional FIT in individuals identified as high‐risk for CRC by a well‐established risk assessment score. The sensitivity of the FIT‐sDNA test for detecting advanced colorectal neoplasia was superior to that of the FIT, especially in detecting advanced precancerous neoplasia (62.4% vs. 30.9%). Correspondingly, the FIT‐sDNA test had significantly higher NPVs than the FIT in both CRC and advanced precancerous neoplasia. For further risk triage in the high‐risk population, sensitivity is the most important factor due to the higher incidence of colorectal neoplasia compared to the average risk population as the priority is to identify more cases and avoid underdiagnosis. In the present study population, the probability of having CRC and advanced colorectal neoplasia with a negative FIT‐sDNA test was 0.4% and 4.5% compared to 1.8% and 8.6% with negative FIT results, respectively. Patients with a negative FIT‐sDNA test are much less likely to have missed CRC or advanced colorectal neoplasia than those with negative FIT results.

Specificity is also important as it determines the number of individuals that may be referred to unnecessary colonoscopies. Currently, the FIT‐sDNA test is recommended by national guidelines for screening the average‐risk population, but it has a potential negative impact with a relatively high false positive rate in the target population.^18^ In the present study, the specificity of the FIT‐sDNA test (87.1%– 90.3%) was inferior to that of the FIT (94.9%–97.2%). A positive FIT‐sDNA test resulted in an increased probability of having CRC from 4.4% to 19.3% compared to 27.6% for the FIT. The probability of having an advanced precancerous lesion with a positive FIT‐sDNA test increased from 8.8% to 26.9% compared to 27.6% for a positive FIT result. The FIT‐sDNA test produced 464 more positive results than the FIT, and it detected 55 more CRC and 122 more advanced precancerous lesions. The detection rate of advanced colorectal neoplasia among the extra FIT‐sDNA test positive results was 38.1%, which was 2.8 times the prevalence rate (13.2%) in the study population. These data suggested that although the FIT‐sDNA test produces more positive results than the FIT, it still minimizes the risk of underdiagnosis and increases the detection rate in a high CRC risk population.

As previously mentioned, the major challenge of preliminary screening using a risk adopted score or the FIT in a limited colonoscopy resource country with a large population are the high positive rate and low detection rate. A recent national wide screening program identified 13% of 1.3 million participants as high‐risk CRC individuals by preliminary screening, but the colonoscopy compliance is only 15%.^16^ Several studies performed repeated FIT that aims to improve the detection rate and effectiveness but with limited success.^20^, ^21^ A recent Chinese expert consensus suggested the potential benefit of the additional FIT‐sDNA test for individuals who are identified as high CRC risk by preliminary screening in a limited colonoscopy resource area.^22^ This is the first study to demonstrate that comparing with FIT, the FIT‐sDNA test is a promising further risk triage modality for individuals who are identified as high CRC risk by preliminary screening. Although an additional FIT may lead to increased detection as well, the FIT‐sDNA test is superior to FIT for individuals identified by preliminary screening due to the following reasons. First, FIT‐sDNA had a higher sensitivity and detected significant more CRC and advanced precancerous neoplasia than FIT. Noticeably, due to the high incidence of colorectal advanced neoplasia among these individuals, one test with higher sensitivity and NPV is more important and fits the situation better to avoid the risk of miss diagnosis. Second, the colonoscopy compliance was higher for the FIT‐sDNA test than FIT. For average‐risk population screening, individuals were more likely to prefer the FIT‐sDNA test over FIT and the colonoscopy compliance after a positive FIT‐sDNA test result has been reported to be higher than after a positive FIT.^23^, ^24^ Therefore, for individuals who have a positive risk‐adapted score or an FIT result in a limited colonoscopy resource area, an additional FIT‐sDNA test can be used to select patients for an urgent follow‐up colonoscopy or provided to individuals who refused colonoscopy to increase the colonoscopy compliance.

The present study had several strengths and limitations. This large‐scale prospective clinical evaluation demonstrated the superior clinical performance of the FIT‐sDNA test compared to the FIT in individuals identified as high CRC risk. Different to prior studies,^16^ we targeted individuals who are at high risk for CRC selected by strict inclusion criteria. In the present study, 186 (4.4%) participants were diagnosed with CRC, and 375 (8.8%) participants were diagnosed with advanced precancerous neoplasia. The prevalence of CRC and advanced precancerous neoplasia was comparable to other studies that targeted individuals with a positive FIT or risk‐adopted score.^8^, ^20^, ^21^ However, limitations include that test intervals and cost‐effectiveness of the FIT‐sDNA test were not analyzed in this study which only focused on the clinical performance of the FIT‐sDNA test among the high CRC risk individuals. Although the sensitivity of the FIT‐sDNA test was superior to the FIT in the present study, some modeling studies suggested that screening with the FIT‐sDNA test every 3 years might not provide a favorable balance of benefits and harms compared with FIT once a year.^6^ However, we need to do more longitude follow‐up studies with larger sample size and collect more information on labor cost and exact colonoscopy resources to analyze test intervals and cost‐effectiveness of the FIT‐sDNA test. Second, since each participant was required to provide a stool sample for the FIT‐sDNA test in our study, accurate data on the high CRC risk individuals’ compliance to the FIT‐sDNA test and factors associated with patient preferences were not available. Further real‐world studies for evaluation of compliance are warranted. In a word, the exact role of the FIT‐sDNA test in CRC screening requires additional investigation beyond the scope of the present study.

In conclusion, the FIT‐sDNA test demonstrated significantly higher sensitivities and NPVs for both CRC and advanced precancerous lesions compared to the FIT alone, indicating its potential role for further risk stratification after preliminary screening in CRC screening.

## 4 MATERIALS AND METHODS

### 4.1 Study design

Colorectal Cancer Early Screening in China (Clear‐C) is a multicenter, prospective clinical study that was performed in eight tertiary‐level hospitals across eight provincial territories in China from September 2018 to November 2019. The eight hospitals contain the Second Affiliated Hospital Zhejiang University School of Medicine which is the leading unit of the trial, the First Affiliated Hospital with Nanjing Medical University, Shanxi Provincial People's Hospital, Tianjin Medical University General Hospital, Hubei Cancer Hospital, West China Hospital of Sichuan University, Fudan University Shanghai Cancer Center, and the First Affiliated Hospital of Zhengzhou University. The Clear‐C study followed the requirements of the Technical Guidelines for Clinical Trials of In Vitro Diagnostic Products from the National Medical Products Administration (NMPA, Reg No. CSZ2000050). The ethics and study approval were granted by the Institutional Review Board at each hospital and the ethic approval number of the Second Affiliated Hospital Zhejiang University School of Medicine was (2018) LSSJ NO. (015). All participants provided written informed consent.

The study was designed by the authors and Beijing Mingze Technology, a contract research organization (CRO), provided data collection and monitoring service. A third‐party statistician analyzed the data with the leading principal investigator.

### 4.2 Study population

The study population was comprised of participants who were identified as high‐risk for CRC by HRFQ which has been widely used as the primary screening method in China and recommended by the Chinese expert consensus on CRC screening and China guidelines for the screening, early detection, and early treatment of CRC.^10^, ^15^, ^22^, ^25^ Experienced doctors interviewed participants to collect information about their exposure to CRC risk factors using HRFQ and all data were recorded in case report forms. Briefly, a high‐risk population was defined as individuals aged 40 and 74 years and who had at least one of the following risk factors^4^, ^22^, ^25^, ^26^, ^27^: (1) family history of CRC (first degree relative); (2) history of positive FOBT but did not undergo a colonoscopy in the past 5 years; and (3) two or more clinical symptoms or conditions, including chronic constipation/diarrhea, mucous/bloody stool, chronic appendicitis or appendectomy, chronic biliary track diseases, and history of psychiatric trauma. The detailed definition of the inclusion criteria is included in the Appendix (Supporting information).

The exclusion criteria were as follows: (1) failure to provide stool samples; (2) history of familial adenomatous polyposis, Crohn's disease, or ulcerative colitis; (3) history of colonoscopy and lesion removal within the previous 5 years; and (4) previous history of confirmed CRC.

### 4.3 Clinical procedures

After providing written informed consent, all eligible participants were required to provide a stool sample for the FIT‐sDNA test and FIT prior to bowel preparation and undergo a colonoscopy in the same hospital within 90 days. Endoscopists followed the guidelines for screening, endoscopic diagnosis, and treatment of early CRC in China.^28^ All colonoscopies were performed by experienced endoscopists without knowing the results of either stool test. Abnormal findings during the colonoscopy were sent for pathology examination. Colonoscopy and tissue biopsy pathology results were used as the gold standard in the present study, and the results were reviewed by the pathologist and investigator at each hospital. If two or more colorectal lesions were diagnosed, only the most advanced lesion and its location were used for classification. The adenoma detection rate was 25% in the present study.

### 4.4 Laboratory procedures

Stool specimens were collected and transferred to the medical laboratory at each hospital. Laboratory technicians were blinded to patient identity, clinical history, and the comparator FIT result of the participants. The FIT‐sDNA test (ColoClear^®^, New Horizon Health Technology, China) was performed according to the manufacturer's instructions, and the details of the stool collection and processing are shown in the Supporting information. Briefly, the stool specimen was pretreated with the sample preparation kit provided by the manufacturer, and the integrated FIT test was performed on the same day of stool specimen collection. The DNA extraction and polymerase chain reaction (PCR) analysis were performed within 7 days of the pretreatment. Alternatively, the stool specimen was frozen at −80°C after finishing the integrated FIT test for subsequent DNA extraction and PCR analysis.

The FIT‐sDNA test is a newly developed assay that consists of a qPCR assay for KRAS mutation, aberrant methylation of the BMP3 promoter region, aberrant methylation of the NDRG4 promoter region, β‐Actin (ACTB), and B2M (reference gene for DNA quantity) as well as a hemoglobin immunoassay. A logistic‐regression algorithm incorporating all the above parameters was used to compute a single composite score with values of 165 or above considered to be positive (Table S4). The detailed information for specimen flow and approach to extraction and analysis of DNA and hemoglobin was shown in Figure S2.

The comparator FIT (FOBT kit (LFD) approved by NMPA, Kangzhu Ltd., China) was performed according to the manufacturer's instructions using the same stool specimen for the FIT‐sDNA test. Laboratory technicians were blinded to identify the patient, clinical history, and the FIT‐sDNA test results. A red line appeared in the test zone of the FIT kit if the sample concentration exceeded 100 ng Hb/mL.

### 4.5 Outcome

The primary outcome was the sensitivity of the FIT‐sDNA test in detecting CRC and the specificity for detecting nonadvanced adenomas or negative colonoscopy findings. The secondary outcome was the sensitivity of the FIT‐sDNA test in detecting advanced precancerous neoplasia. The CRC stage was determined according to the 8th edition of Cancer Staging Manual from American Joint Committee on Cancer (AJCC). The definition of advanced adenomas was adenomas with either high‐grade intraepithelial neoplasia, villous elements, or measuring ≥1 cm. Advanced precancerous neoplasia was defined as advanced adenomas or traditional sessile adenomas and sessile serrated adenomas/polyps ≥1 cm.

### 4.6 Statistical analysis

The sample size calculation was based on the hypothesis that the sensitivity of the FIT‐sDNA test in detecting CRC is no less than 65% and that the specificity of the FIT‐sDNA test is no less than 75%. According to the data of the preclinical study submitted to NMPA (conducted by Prof. Shu Zheng and New Horizon Health Technology), the expected sensitivity in detecting CRC was set as 80%. At a two‐sided type I error rate of 0.05 and power of 80%, the minimum number required for CRC and patients with nonadvanced adenomas or negative colonoscopy findings was 72 and 563, respectively. The secondary hypothesis was that the sensitivity of the FIT‐sDNA test in detecting advanced precancerous neoplasia is superior to the FIT. To test the secondary hypothesis, the expected sensitivities of the FIT‐sDNA test and comparator FIT in detecting advanced precancerous neoplasia were 40% and 25%, respectively. To demonstrate superiority, the required minimal number of valid patients with advanced precancerous neoplasia was 152 with a power of 80% and type I error rate of 0.05. Considering the dropout and loss rate during the clinical study, the required sample sizes for patients with CRC and advanced precancerous neoplasia were 90 and 180, respectively. For patients with nonadvanced adenomas or negative colonoscopy findings, the required sample size was 700. In the present study, we found 186 participants with CRC, 375 participants with advanced precancerous neoplasia, and 3,684 participants with nonadvanced adenomas or negative colonoscopy findings in the final analysis, therefore fulfilling the required sample size for testing the research hypotheses.

Sensitivity and specificity with 95% CIs were used to assess the clinical performance for detecting advanced colorectal neoplasia. The McNemar paired‐comparison test was used to compare the outcome difference between groups or tests. All analyses were performed using SPSS 22 (International Business Machines Corporation, New York City, the United States of America).

## AUTHOR CONTRIBUTIONS

Ke‐Feng Ding, Yi‐You Chen, Ning Lv, Shu Zheng, and Yan‐Hong Gu conceived and designed the study. Ye‐Ting Hu, Xiao‐Feng Chen, Chun‐Bao Zhai, Gang Liu, Zhi‐Guo Xiong, Zi‐Qiang Wang, San‐Jun Cai, Wen‐Cai Li, Cai‐Hua Wang, and Zhi‐Hua Tao contributed to the recruitment of participants. Xiang‐Xing Kong, Qian Xiao, Guo‐Zhong Jiang, Li‐Yun Niu, Jian‐Long Men, Qing Wang, Shao‐Zhong Wei, Jun‐Jie Hu, Ting‐Han Yang, and Jun‐Jie Peng led the data collection. Ye‐Ting Hu, Xiao‐Feng Chen, and Xiao‐Tian Yu contributed to the data analysis and data interpretation. Ye‐Ting Hu, Xiao‐Tian Yu, and Ke‐Feng Ding drafted the manuscript. All authors provided critical review and final approval of the manuscript. The corresponding author attests that all listed authors meet authorship criteria and that no others meeting the criteria have been omitted.

## CONFLICT OF INTEREST STATEMENT

Xiao‐Tian Yu is the Medical Director, Ning Lv is the Chief Technology Officer, and Yi‐You Chen is the Chief Scientist at Hangzhou New Horizon Health Technology Company. The other authors declare no conflicts of interest.

## ETHICS STATEMENT

The Clinical Trials registration number of the study was NCT04287335. The study was carried out on the basis of the guideline of the Declaration of Helsinki and was approved by the Ethics Committees of the Second Affiliated Hospital Zhejiang University School of Medicine, the First Affiliated Hospital with Nanjing Medical University, Shanxi Provincial People's Hospital, Tianjin Medical University General Hospital, Hubei Cancer Hospital, West China Hospital of Sichuan University, Fudan University Shanghai Cancer Center, and the First Affiliated Hospital of Zhengzhou University. The ethic approval number of the Second Affiliated Hospital Zhejiang University School of Medicine was (2018) LSSJ NO. (015). Written informed consent was obtained from all participants.

## Supporting information

Supporting Information

## Data Availability

The clinical data generated in this study are available within the article and its Supporting information. Additional materials and methods are included in Supporting information. Other detailed data in this study are available upon reasonable request from the corresponding author.
