# Supplementary material for: Clinical evaluation of a multitarget fecal immunochemical test‐sDNA test for colorectal cancer screening in a high‐risk population: a prospective, multicenter clinical study
Source: MedComm (2020). 2023 Aug 12;4(4):e345. doi: 10.1002/mco2.345 (PMC10422070; doi:10.1002/mco2.345)
Supplement: Supplementary file 1 — Supporting Information [file MCO2-4-e345-s001.docx]

**Clinical evaluation of a multitarget FIT-sDNA test for colorectal cancer screening in a high-risk population: a prospective, multicenter clinical study**

Ye-Ting Hu^1,14#^, Xiao-Feng Chen^2#^, Chun-Bao Zhai^3#^, Xiao-Tian Yu^4#^, Gang Liu^5^, Zhi-Guo Xiong^6,7^, Zi-Qiang Wang^8^, San-Jun Cai^9^, Wen-Cai Li^10^, Xiang-Xing Kong^1,14^, Qian Xiao^1,14^, Cai-Hua Wang^11^, Zhi-Hua Tao^12^, Li-Yun Niu^3^, Jian-Long Men^5^, Qing Wang^5^, Shao-Zhong Wei^6,7^, Jun-Jie Hu^6,7^, Ting-Han Yang^8^, Jun-Jie Peng^9^, Guo-Zhong Jiang^10^, Ning Lv^4^, Yi-You Chen^4^, Shu Zheng^13^, Yan-Hong Gu^2*^, Ke-Feng Ding^1,14*^

1. Department of Colorectal Surgery and Oncology Key Laboratory of Cancer Prevention and Intervention, China National Ministry of Education, Key Laboratory of Molecular Biology in Medical Sciences, Zhejiang Province, China, The Second Affiliated Hospital, Zhejiang University School of Medicine, Hangzhou, Zhejiang, China; Zhejiang Provincial Clinical Research Center for CANCER

2. Department of Oncology, The First Affiliated Hospital with Nanjing Medical University (Jiangsu Province Hospital), Nanjing, China.

3. Department of Anorectal Surgery, Shanxi Provincial People's Hospital, Taiyuan, China.

4. Hangzhou New Horizon Health Technology Co., Ltd., Hangzhou, China.

5. Department of General Surgery, Tianjin Medical University General Hospital, Tianjin, China.

6. Department of Gastrointestinal surgery, Hubei Cancer Hospital, Wuhan, China.

7. Colorectal Cancer Medical Research Center of Hubei, Wuhan, China.

8. Department of Gastrointestinal Surgery, West China Hospital of Sichuan University, Chengdu, China.

9. Department of Colorectal Surgery, Fudan University Shanghai Cancer Center, Shanghai, China.

10. Department of Pathology, The First Affiliated Hospital of Zhengzhou University, Zhengzhou, China.

11. Department of Gastroenterology, The Second Affiliated Hospital of Zhejiang University School of Medicine, Hangzhou, China.

12. Department of Clinical Laboratory, The Second Affiliated Hospital of Zhejiang University School of Medicine, Hangzhou, China.

13.Cancer Institute, Key Laboratory of Cancer Prevention and Intervention, Ministry of Education, The Second Affiliated Hospital, Zhejiang University School of Medicine, Hangzhou, China.

14. Zhejiang University Cancer Center, Hangzhou, China.

#These authors contributed equally to this work

*Co-corresponding author

**Corresponding author:**

Dr. Ke-Feng Ding, Department of Colorectal Surgery and Oncology, Key Laboratory of Cancer Prevention and Intervention, Ministry of Education, The Second Affiliated Hospital, Zhejiang University School of Medicine, Hangzhou, 310000, [dingkefeng@zju.edu.cn](mailto:dingkefeng@zju.edu.cn)

Dr. Yan-Hong Gu, Department of Oncology, The First Affiliated Hospital with Nanjing Medical University (Jiangsu Province Hospital), Nanjing, 210000, [guluer@163.com](mailto:guluer@163.com)

**Supplementary Appendix**

**Table of Contents**

[Table S1. Characteristics of Clinical Findings. 4](#_Toc135837087)

[Table S2. Numbers of Participants Required to Be Screened with Colonoscopy, Multitarget FIT-sDNA Test, and FIT to Detect One Colorectal Cancer and One Advanced Precancerous Neoplasia. 5](#_Toc135837088)

[Table S3. Sensitivity and specificity of the Multitarget FIT-sDNA Test and FIT in different subgroups. 6](#_Toc135837089)

[Figure S1. Receiver Operating Characteristic (ROC) Curves Comparing Multitarget FIT-sDNA Testing for the Detection of Colorectal Cancer and advanced precancerous neoplasia. 7](#_Toc135837090)

[Figure S2. Study Specimen Flow and Approach to Extraction and Analysis of Stool DNA and Hemoglobin. 8](#_Toc135837091)

[Development of analysis algorithm for combined test 10](#_Toc135837092)

[Overview 10](#_Toc135837093)

[Calculation 10](#_Toc135837094)

[Table S4. ColoClear Multi-target sDNA Algorithm – Score Calculation 12](#_Toc135837095)

[The specific definition of inclusion criteria in this study 13](#_Toc135837096)

[Fecal sample collection - DNA extraction and purification - sulfite transformation 14](#_Toc135837097)

[Stool sample collection 14](#_Toc135837098)

[DNA extraction and purification 14](#_Toc135837099)

[DNA sulfite transformation 16](#_Toc135837100)

# Table S1. Characteristics of Clinical Findings.

| **Clinical characteristics** | **Colorectal cancer**  **(N=186)** | **Advanced precancerous neoplasia ***  **(N=375)** |
| --- | --- | --- |
| CRC Stage†, n (%) |  |  |
| Ⅰ | 32(17.2) | / |
| Ⅱ | 53(28.5) | / |
| Ⅲ | 55(29.6) | / |
| Ⅳ | 18(9.7) | / |
| Unknown | 28(15.1) | / |
| Location, n (%) |  |  |
| Proximal (≥60cm, Right hemicolon) | 32(17.2) | 85(22.7) |
| Distal  (<60cm, Left hemicolon) | 154(82.8) | 251(66.9) |
| Unknown | / | 39(10.4) |

*****Advanced precancerous neoplasia include advanced adenomas , SSA/P andTSA≥ 1cm)

†These stages of colorectal cancer, as defined by the system recommended by the American Joint Committee on Cancer (TNM 8^th^ edition)

# Table S2. Numbers of Participants Required to Be Screened with Colonoscopy, Multitarget FIT-sDNA Test, and FIT to Detect One Colorectal Cancer and One Advanced Precancerous Neoplasia.

| **Finding** | **Number Needed to Screen (95% CI)** | | | | |
| --- | --- | --- | --- | --- | --- |
|  | Colonoscopy |  | FIT-sDNA Test |  | FIT |
| Any colorectal cancer | 23(20-26) |  | 25(21-29) |  | 37(30-43) |
| Stage I to Ⅲ colorectal cancer | 39(32-48) |  | 41(33-50) |  | 58(45-71) |
| Advanced precancerous neoplasia | 11(10-13) |  | 18(16-20) |  | 37(30-43) |

# Table S3. Sensitivity and specificity of the Multitarget FIT-sDNA Test and FIT in different subgroups.

|  | **CRC/AA**  **NO.** | | **FIT-sDNA**  **Sensitivity(%) (95%CI)** | **FIT**  **Sensitivity(%) (95%CI)** | **FIT-sDNA**  **Specificity(%) (95%CI)** | **FIT**  **Specificity(%) (95%CI)** |
| --- | --- | --- | --- | --- | --- | --- |
| **Total samples (N=4245)** | | CRC (N=186) | 91.9 (86.8-95.3) | 62.4  (55.2-69.0) | 87.1 (85.9-88.1) | 94.9  (94.1 - 95.5) |
|  |  | AA (N=375) | 63.5 (58.3-68.3) | 30.1  (26.4-35.8) |  |  |
| **History of positive FOB test (N=182)** | | CRC (N=9) | 100.0 (62.9-100.0) | 77.8  (45.3-93.7) | 80.9 (73.6-86.7) | 88.8  (82.8 - 92.9) |
|  |  | AA (N=21) | 76.2 (52.5-90.9) | 61.9  (40.9-79.2) |  |  |
| **Family history of CRC (N=376)** | | CRC (N=2) | 100.0 (19.8-100.0) | 50.0  (9.5-90.6) | 88.2 (84.1-91.3) | 97.6  (95.4 - 98.9) |
|  |  | AA (N=36) | 55.6 (38.3-71.7) | 25.0  (13.8-41.1) |  |  |
| **Clinical symptoms or conditions (N=3632)** | | CRC (N=172) | 91.3 （85.8-94.9） | 61.6  (54.2-68.6) | 87.3 （86.1-88.5） | 94.8  (94.0 - 95.6) |
|  |  | AA (N=314) | 63.4 (57.8-68.7) | 29.3  (24.5-34.6) |  |  |
| **Mixed indications  (N=55)** | | CRC  (N=3) | 100  (31.0-100) | 66.7  (12.5-98.2) | 76.9  (62.8-87.0) | 92.2  (80.3-97.5) |
|  |  | AA  (N=4) | 75.0  (21.9-98.7) | 50.0  (9.2-90.8) |  |  |


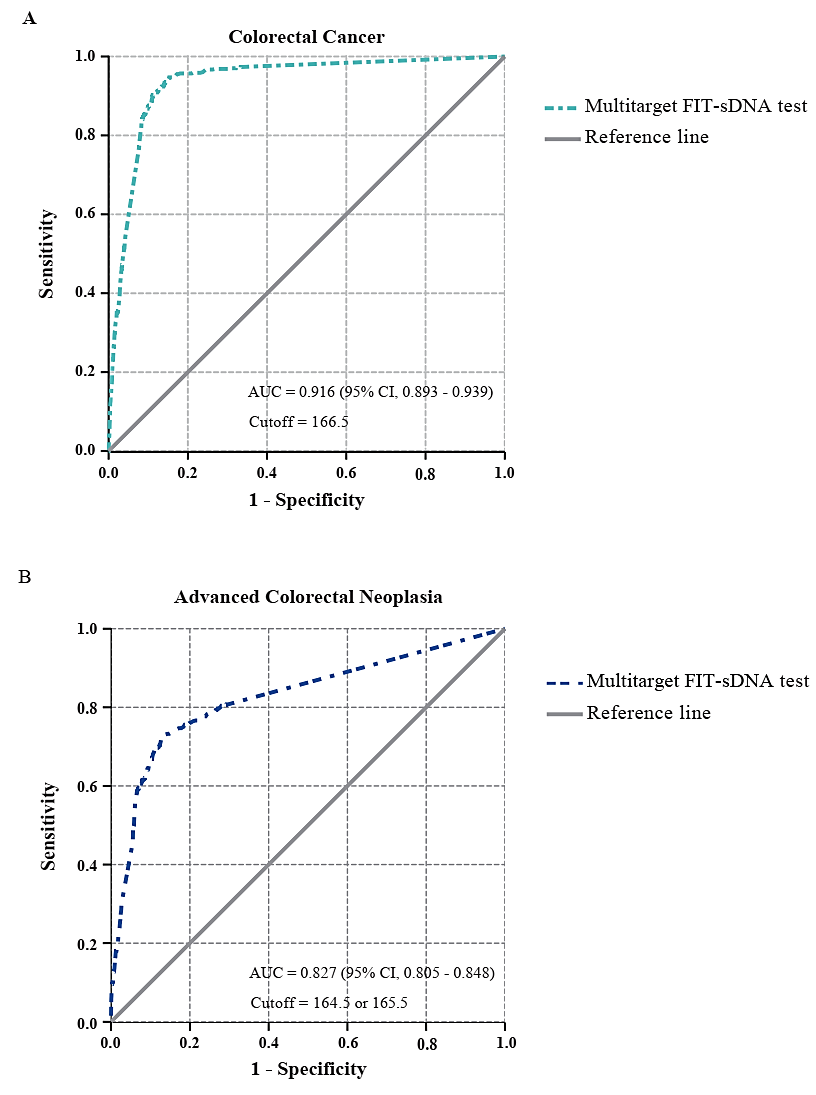


# Figure S1. Receiver Operating Characteristic (ROC) Curves Comparing Multitarget FIT-sDNA Testing for the Detection of Colorectal Cancer and advanced precancerous neoplasia.

ROC curves for the Multitarget stool FIT-DNA test for the detection of colorectal cancer (Panel A) and advanced colorectal neoplasia (Panel B). For colorectal cancer, the area under the ROC curve was 0.916 for the FIT-DNA test (95% confidence interval [CI] for the difference in area, 0.893 to 0.939). For advanced colorectal neoplasia, the area under the ROC curve was 0.827 for the DNA test (95% CI for the difference in area, 0.805 to 0.848).

**
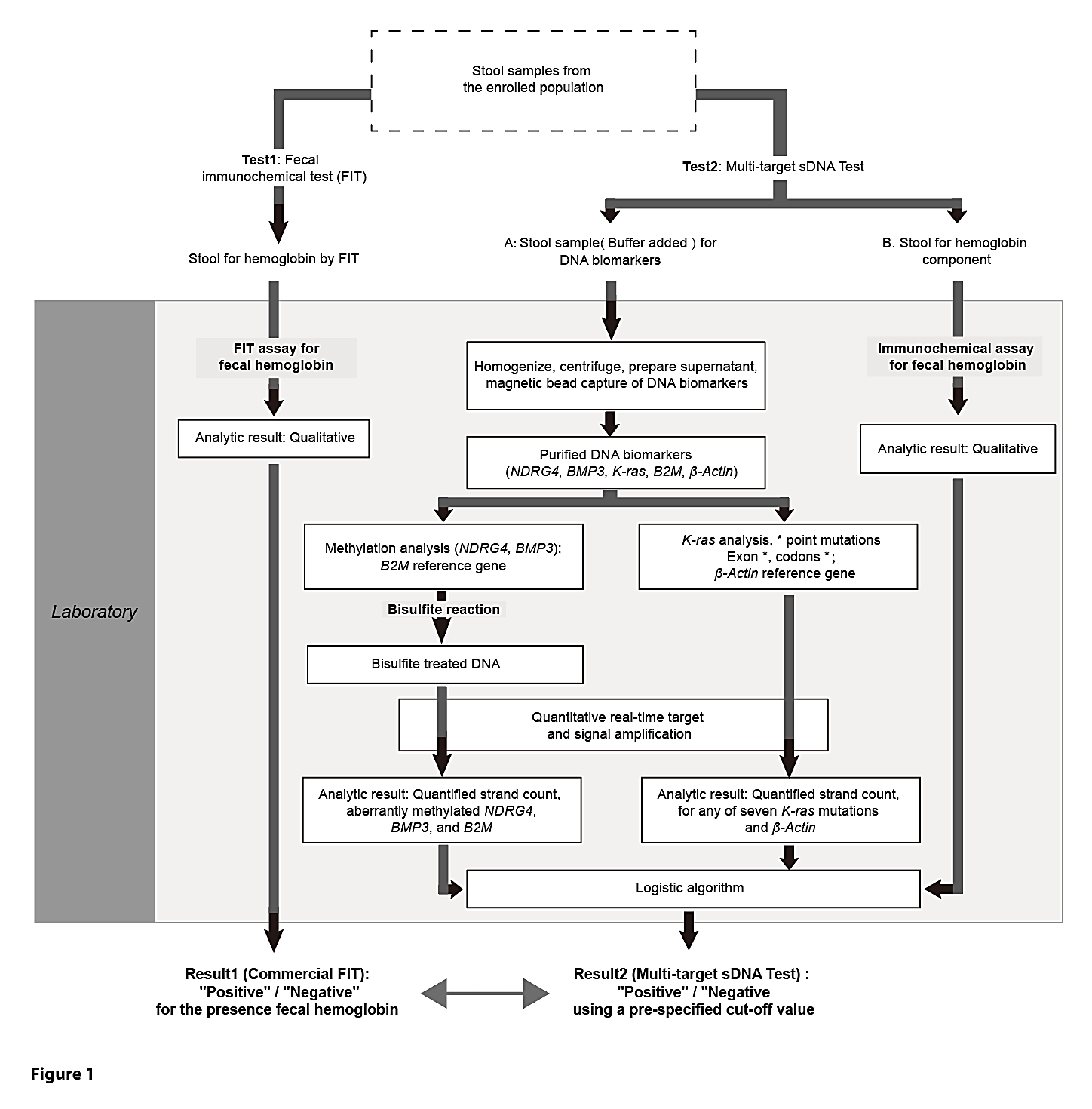
**

# Figure S2. Study Specimen Flow and Approach to Extraction and Analysis of Stool DNA and Hemoglobin.

Study enrolled populations passed a single spontaneous stool sample into provided collection containers, sampled the stool for the commercial FIT test (Test 1) and for the DNA biomarkers(A) and hemoglobin component(B) of the multi-target stool DNA test (Test 2). All three samples were returned to the laboratory together for blinded analyses. The commercial FIT test was performed per manufacturer’s instructions which provides a qualitative result of positive or negative for the presence of fecal hemoglobin based on a quantitative result. The hemoglobin component of the multi-target sDNA test was analyzed by a separate immunochemical assay. The buffered stool sample(A) was normalized with additional buffer and mixed and centrifuged. Sequence specific DNA biomarker targets were isolated from the supernatant using a magnetic bead method. The extracted DNA was split, and one portion was subjected to the bisulfite reaction for the identification of aberrantly methylated *NDRG4* and *BMP3* and the other portion was used to determine the presence of any of seven *K-ras* point mutations. The test does not distinguish the specific mutation. Using quantitative real-time target and signal amplification, *B2M* for total DNA content and methylated *NDRG4* and *BMP3* and were analyzed in one well of a 96 well PCR plate while *β-Actin* for total DNA content and 7 *K-ras* point mutations were analyzed in a second well of the same plate. The results of this analysis for methylated *NDRG4*, methylated *BMP3*, mutated *K-ras*, *B2M*, *β-Actin* in log strands was combined with the results of the stool DNA test hemoglobin component in a pre-specified logistic regression algorithm, the output of which determined if the stool DNA test was positive, if the pre-specified cut-off value was ≥ 165, or negative.

# Development of analysis algorithm for combined test

## Overview

The algorithm and all supported analysis methods were submitted to the National Medical Products Administration (NMPA) of China on February 26, 2020 as part of the registration materials for the *KRAS* gene mutation *BMP3/NDRG4* gene methylation and fecal occult blood joint detection kit. The algorithm and weighting factors were locked in the software on April 12, 2017. The research sample test started in October 2018. All the results given in the research are calculated based on this algorithm.

The basic form of the calculation formula is $Score=\left[ \frac{e^{K}}{\left( 1+e^{K} \right)}+E*\left( F-KRAS \right) \right]*G$ where K = S + A × (FOBT positive or negative) + B × (*KRAS* Ct) + C × (*BMP3* Ct) + D × (*NDRG4* Ct). S,G E and F are constants, and A, B, C, and D are the coefficients used in the comprehensive calculation of the four individual test results. These parameters were established using a training data set and a validation data set. The purpose is to obtain the best coefficient when the related loss function (Loss Function) is minimized through machine learning.

The Score calculated by formula for the test reflects the probability that each test sample is obtained from a subject who has colorectal cancer or advanced adenoma (positive), versus a normal subject (negative). The cutoff value for the method is 165. That is, when the Score value is less than 165, the sample result is negative, and when the Score value is equal to or greater than 165, the sample result is positive.

## Calculation

**Step 1.** Confirm whether the results are valid. The internal reference *ACTB* gene is used for the *KRAS* reaction. If the *ACT*B result is negative, then the *KRAS* test defined as invalid; the *BMP3/NDRG4* methylation target reaction uses the *B2M* gene as internal reference. If the *B2M* result is negative, the *BMP3/NDRG4* methylation target reaction is invalid;

**Step 2.** Substitute the *KRAS* gene mutation Ct value, *BMP3* gene methylation Ct value, *NDRG4* gene methylation Ct value and fecal occult blood test result (positive or negative) into the calculation formula to calculate the K value and Logistic regression value.

**Step 3.** Generating the final call for the test. If the Score value is less than 165, the sample result is negative. If the Score value is equal to or greater than 165, the sample result is positive.

# Table S4. ColoClear Multi-target sDNA Algorithm – Score Calculation

|  |  | *KRAS* mutation marker | Methylation markers | | Human hemoglobin |
| --- | --- | --- | --- | --- | --- |
| Inputs to test score calculation | Ct value or fecal hemoglobin | *KRAS* Ct | *BMP3* Ct | *NDRG4* Ct | FOBT  （positive or negative） |
|  | Logistic Score formula | K=S+FOBT×（A）+*KRAS*×（B）+*BMP3*×（C）+*NDRG4*×（D） | | | |
| Test Score calculations | Logistic Score formula | $Score=\left[ \frac{e^{K}}{\left( 1+e^{K} \right)}+E*\left( F-KRAS \right) \right]*G$ | | | |
| Multi-target stool DNA  Result determination | Reference range:  Negative＜165 | If the Composite Score＜165，result as “Negative”; If the Composite Score≥165，result as “Positive”; | | | |

# The specific definition of inclusion criteria in this study

Chronic constipation refers to male who defecates less than three times per week or female who defecates less than twice per week. Or defecate difficult, small defecate quantity, medical history more than two years and more than two episodes per year. Chronic diarrhea means loose stools or defecate more than twice per day or diarrhea more than three months in recent two years or defecate last more than one week each time. Mucous and bloody stool means the presence of visible mucus or blood in the feces. Chronic appendicitis should be formally diagnosed by a secondary hospital or above, and the onset time should be at least 1 year. The time of appendectomy should be at least 1 year from the investigation time. Chronic biliary track diseases refer to the history of chronic cholecystitis or cholecystectomy for more than 1 year. The history of psychiatric trauma refers to the great changes in family or personal life in the past 20 years, such as the death of relatives, career failure, divorce, etc. The individual suffers from great psychological stress or in a state of long-term depression.

# Fecal sample collection - DNA extraction and purification - sulfite transformation

## Stool sample collection

**Step 1.** Fecal samples were collected by the fecal test pretreatment kit of Hangzhou New Horizon Health Technology Co., Ltd;

**Step 2.** Use the plastic sampling spoon provided by the kit to take a spoonful of feces (equivalent to the size of the first joint of thumb, about 5g), and put it into the sample tube A together with the sampling spoon The total volume after sampling should not exceed 25ml (the sample tube has a scale mark);

**Step 3.** Take out the sample tube B (including desiccant) and pull out the tube cover. Use the sampling rod to continuously puncture and sample in 5 different parts of feces, and then insert the tube cover back into the sample tube B to cover tightly;

**Step 4.** After sampling, tighten the two sample tubes and put them into the self sealing bag respectively, and then put them into the green inner box. Pay attention to the preservation of fecal samples: fecal samples are recommended to be stored at room temperature for no more than 7 days; for long-term preservation, it is recommended to store below - 60℃ for no more than 1 month (only for sample tube A).

## DNA extraction and purification

**Step 1.** Shake and mix sample a tube well for 30sec, incubate overnight at 50 ℃ or at 65 ℃ for 10min, centrifuge at 5000rpm for 10min, and transfer 9ml supernatant to another new 50ml centrifuge tube;

**Step 2.** Add 1ml extraction auxiliary solution, 60 μl magnetic bead solution and 10ml isopropanol, vortex oscillation for 10sec, incubate at 65℃ for 20min, during the incubation period, mix it upside down every 5min, after the incubation, put the centrifuge tube on the magnetic frame, stand for 3min, wait for the magnetic bead fully adsorbed on the tube wall, discard the waste liquid;

**Step 3.** Add 12ml of washing solution, vortex oscillation until the magnetic beads fall off the pipe wall, and let it stand for 3min. Then put it into the magnetic frame again, and let it stand for 3min. When the magnetic beads are fully adsorbed on the pipe wall, discard the waste liquid;

**Step 4.** Add 15ml 80% ethanol solution, vortex oscillation until the magnetic beads fall off from the tube wall, and let it stand for 3min, then put it into the magnetic frame, and let it stand for 3min. When the magnetic beads are fully adsorbed on the tube wall, discard the waste liquid, and repeat this step once;

**Step 5.** Use a pipette to suck up the residual liquid at the bottom, open the cover, incubate the centrifuge tube at 65℃ for 5min, take out the magnetic beads after drying, add 1.5ml preheated eluent I, use a 1000 μl pipette to purge the magnetic beads from the tube wall, pump repeatedly, transfer them together with the magnetic beads to 2ml centrifuge tube, close the centrifuge tube cover and incubate at 65℃ for 5min;

**Step 6.** After centrifugation at 13000 rpm for 3 min, 600μl supernatant was transferred to a new 1.5 ml centrifuge tube, 600μl column binding solution was added, and the mixture was well mixed. 600μl of the above mixture was transferred to the DNA purification column, centrifuged at 13000 rpm for 1 min, and the waste liquid was discarded, and this step was repeated once;

**Step 7.** Add 600μl 90% ethanol solution into the DNA purification column, centrifuge at 13000 rpm for 1 min, discard the waste liquid and repeat twice;

**Step 8.** After centrifugation at 13000rpm for 3min, the DNA purification column was put into a new 1.5ml centrifuge tube, the cover of the centrifuge tube was opened, incubated at 65℃ for 5min, and dried;

**Step 9.** Add 100 μl preheated eluent II to the middle of the DNA purification column, close the cover, incubate at 65℃ for 5 min, and centrifuge at 13000 rpm for 2 min to obtain the eluted DNA solution;

**Step 10.** UV spectrophotometer was used to detect the concentration and DNA quality. The concentration of DNA should be more than 50 ng/μl, and the od260/280 of DNA should be between 1.6 and 2.0. Pay attention to the preservation of DNA samples: if the extracted DNA is stored for a long time at - 25 ~ - 15℃ for no more than 6 months, the transformed DNA is recommended to be stored at - 25 ~ - 15℃ for no more than 2.5 months, and the DNA is repeatedly frozen and thawed for no more than 3 times.

## DNA sulfite transformation

**Step 1.** Transfer 40 μl extracted DNA solution to 1.5ml centrifuge tube, add 4 μl 3M NaOH (the diluted denaturation solution), and incubate in 42℃ bath for 20min;

**Step 2.** Add 200 μl conversion solution, mix well, and incubate at 50℃ for 16 hours;

**Step 3.** Add 550μl column binding solution, mix well, transfer to DNA purification column, centrifuge at 13000 rpm for 2 min, discard the waste liquid, centrifuge again for 3 min, discard the waste liquid; note that after this step, a new collection tube needs to be replaced, and if there is liquid residue in the purification column, use a 10 μl gun head to absorb the residual liquid.

**Step 4.** Add 600μl 90% ethanol to the DNA purification column, centrifuge at 13000 rpm for 2 min, discard the waste liquid and centrifuge again for 15 sec;

**Step 5.** Add 300 μl desulfurizing solution (90% ethanol solution of 0.02M NaOH), place at room temperature (15-30℃) for 30min, centrifuge at 13000rpm for 2min, and discard the waste liquid;

**Step 6.** Add 600 μl 90% ethanol, centrifuge at 13000 rpm for 2 min, discard the waste liquid, repeat this step once, and then centrifuge again for 3 min;

**Step 7.** The DNA purification column was put into a new 1.5ml centrifuge tube, 40 μl eluent was added, incubated in 50℃ oven or metal bath for 10min, centrifuged at 13000rpm for 2min, and the transformed DNA solution was obtained and stored at - 25 ～ - 15℃ for standby.
